# The influence of land use in the catchment area of small waterbodies on the quality of water and plant species composition

**DOI:** 10.1038/s41598-022-11115-w

**Published:** 2022-05-04

**Authors:** Barbara Szpakowska, Dariusz Świerk, Anna Dudzińska, Maria Pajchrowska, Ryszard Gołdyn

**Affiliations:** 1 Department of Landscape Architecture, Poznań University of Life Sciences, Dąbrowskiego 159, 60-594 Poznań, Poland; 2 Department of Water Protection, Faculty of Biology, Adam Mickiewicz University, Uniwersytetu Poznańskiego 6, 61-614 Poznań, Poland

**Keywords:** Environmental sciences, Environmental impact, Riparian ecology

## Abstract

Land use significantly affects the function of waterbodies in landscape. Although there have been numerous studies on the influence of the catchment area on the trophic and ecological status of waterbodies, still is not reached an agreement on the width of the buffer zone that is necessary for effective protection of waterbodies. The aim of the study was to show whether small waterbodies are predominantly influenced by land use in the entire catchment area or only in the zone extending 100 or 200 m away from the shoreline. For two years the waters in six small waterbodies located in the Wielkopolska region (Poland) were analysed. The canonical correspondence analysis (CCA) showed that the influence of land use, especially agricultural land, was much greater within the zone extending 100 m from the shore line of the waterbodies than in the total catchment area. Therefore, it would be advisable to move the border of intensive farming areas beyond the entire 100-m-wide buffer zone, or at least to reduce the intensity of agriculture and to introduce diversified and perennial vegetation creating effective biogeochemical barriers.

## Introduction

Waterbodies are important elements of landscape. They differ in size, depth, origin, the ecological status, supply of water, and form of use. Some of them like ponds (the most numerous representatives of inland standing waters) are called small waterbodies. They have water surface area ranging from 1 m^2^ to 2–5 ha and they are up to 2–3 m deep. Small waterbodies significantly influence the local water circulation^1^. They are important because they retain the excess of water in spring. Therefore, they are part of the flood protection and drought prevention strategy, which meets the guidelines of the Water Framework Directive^2^. The concentration of nutrients and the physical parameters of small waterbodies are characterised by high variability because they are considerably influenced by the surrounding land and atmosphere, and usually there is little or no horizontal exchange of water, which causes the accumulation of pollutants^3^. Even in adjacent waterbodies these parameters may differently affect primary producers, including microphytes and macrophytes, which are functional elements of aquatic ecosystems^4^.

The regional effects associated with intensive land use are very typical for decreasing macrophyte diversity^5^. Macrophytes significantly affect the formation of habitat^6,7^. Microphytes quickly react to changes in environmental factors due to their intensive metabolism and short reproduction time. The processes and relationships between them significantly influence the function of the entire aquatic ecosystem^8^. Aquatic plants can be used as bioindicators because it reacts to changes in the environment and helps to predict changes in the ecosystems^9^.

Areas adjacent to waterbodies significantly influence the function of small aquatic ecosystems^10^. Depending on the nature of these areas, they either accelerate or inhibit the inflow of mineral and organic matter to waterbodies^11^. Therefore, the amount of pollutants entering water is primarily related to the type of catchment area^12^. According to Mozgawa^13^, we can distinguish three types of land cover in the catchment area with different barrier-forming properties. Swamps and wetlands are the strongest barriers; forests, meadows, and pastures are moderate barriers, whereas arable land and urbanised areas are zero barriers. Agricultural land is considered to be the most common type of catchment area. It is characterised by increased leaching of nutrients from soil. If 40% of the catchment area is covered by wetlands and waterbodies, it retains over 90% of agricultural contamination^14^. It is through surface runoff from the catchment area that most of the chemical compounds, including nutrients, enter the aquatic environment. This phenomenon is seasonally variable and it is very closely related to climatic conditions, especially rainfall. Apart from that, the outflow of nutrients can be modified by the landform, type of soil, and intensity of the catchment area management^15^.

Although many authors have studied the influence of the catchment area on the trophic and ecological state of waterbodies^16–18^, there is still no agreement on the width of the buffer zone which should be taken into account when assessing this influence, especially for effective protection of waterbodies. According to Novikmec et al.^19^, the entire catchment area of a body of water should be taken into consideration because a narrow buffer zone does not provide sufficient protection. Dudzińska et al.^20^ took zones of 500 and 1000 m into account and concluded that the 500-m-wide zone was sufficiently wide to assess the influence of the catchment area on the waterbody. According to Wilk-Woźniak et al.^11^, the buffer zone should depend on the size of the waterbody. The researchers analysed a zone extending 200 m from the shoreline of small waterbodies (with an area up to 2599 m^2^) and a zone of 400 m for larger ones (with an area of 2600–10,000 m^2^). They found that if there were forests or meadows around, these buffer zone widths were sufficient to protect the waterbodies and ensure the growth of aquatic vegetation. Szpakowska^21^ concluded that a shoreline tree zone which is at least about 20 m wide can reduce the amount of nutrients flowing from fields by 70–80%. Wysocka-Czubaszek and Banaszuk^22^ found that a 6-m-wide strip of grasses and sedges reduced the content of nitrogen in water by 47%, whereas a twenty-meter-wide strip reduced the content of this element by almost 100%.

The analysis of the catchment areas of the waterbodies included in our study showed that they were usually small or asymmetrical, and their boundaries ran at different distances from the shoreline. Therefore, the aim of our study was to check how the quality of water and macrophyte species composition in the waterbodies and their close vicinity was affected by the use of entire catchment areas, and how they were affected by the buffer zones extending 200 m and 100 m away from the shoreline. We hypothesized that the quality of water and vegetation in waterbodies was influenced equally by the use of the entire catchment area. The opposite hypothesis was that this influence is mainly exerted by the zone located close to the shoreline. Our objective was not take into account entire areas of the buffer zones, but only those parts located within the catchment area of the waterbodies under study, which may have a potential impact on the water quality of the waterbodies. However, the distance from individual potential point sources of pollution was also analysed, although they were often located outside the waterbodies catchment area, for its possible influence on the waterbodies.

## Materials and methods

### Characteristics of waterbodies

**Figure 1:**
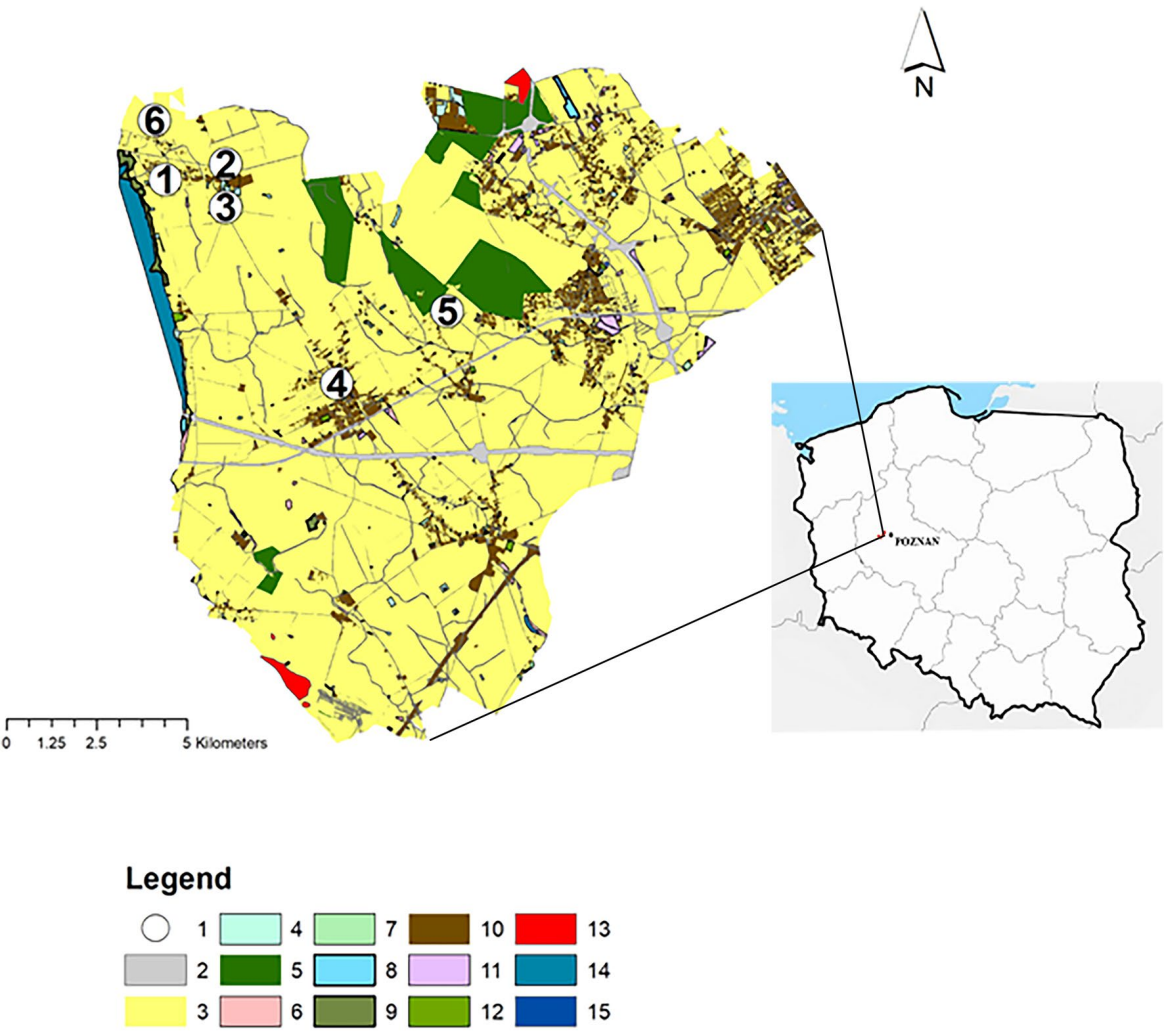
The location of the waterbodies (according to Szpakowska et al.^23^, changed). The map was generated by ArcGIS 10.6. https://support.esri.com/en/Products/Desktop/arcgis-desktop/arcmap/10-6-1#overview. Explanations: 1—waterbodies, 2—roads, 3—arable land, 4—bushy land, 5—forests, 6—wasteland, 7—orchards, 8—ponds, 9—meadows, 10—built-up areas, 11—industrial areas, 12—green areas, 13—services, 14—flowing waters, 15—standing waters.

The study was conducted on six small waterbodies, which were analysed for two years at monthly intervals. The waterbodies were located in the agricultural landscape of the commune of Dopiewo, in the Wielkopolska region in western Poland. They differed in size, catchment area, and origin (Fig. 1, Table 1).

**Table 1:** The characteristics of the waterbodies (according to Szpakowska et al.^23^, changed).

Water- body	Location coordinates	Origin	Length [m]	Wi dth [m]	Depth max [m]	Surface [m^2^]	Volume [m^3^]	Catchment area [ha]	Schindler's coefficient
No. 1	52° 23′ 45″ N 16° 36′ 56″ E	Natural	33.0	13.0	1.0	196	225	0.96	45
No. 2	52° 39′ 50″ N 16° 38′ 25″ E	Natural	38.5	13.7	1.2	497	398	3.54	95
No. 3	52° 23′ 3″ N 16° 38′ 46″ E	Man-made	55.1	35.0	1.7	1929	1716	53.21	319
No. 4	52° 21′ 39″ N 16° 40′ 23″ E	Man-made	158.3	46.8	2.0	5812	7748	112.48	144
No. 5	52° 22′ 21″ N 16° 41′ 52″ E	Natural	20.9	15.6	0.9	256	154	4.67	284
No. 6	52° 24′ 22″ N 16° 37′ 9″ E	Man-made	23.0	16.5	1.9	298	377	2.58	121

Waterbody No. 1 is natural, astatic, the smallest in size and with the smallest catchment area. Waterbody No. 2 is also natural and shallow. Its catchment area is smaller than 4 ha, but it is more than three times larger than the catchment area of waterbody No. 1. It is located in a large post-manorial park. Waterbody No. 3 is located right next to the main road, in the centre of a village. It is the second largest waterbody in terms of volume. It is used for fire fighting and recreational purposes. Before our study sediments were dredged from the bottom of this waterbody. Waterbody No. 4 has the largest surface and catchment areas, the greatest depth and volume of water. It is located in the centre of the large village of Dopiewo. It was the only flow-through waterbody in the group under study. It is regularly shaped and its shores are reinforced with fascine. Like waterbody No. 3, it is used for fire fighting and recreation. Additionally, it is used for angling. Every autumn helophytes are dredged from this waterbody. Waterbody No. 5 is a natural field pond of similar size to waterbody No. 6. Together with its wide belt of reeds, it is a refuge for game, mainly wild boars. Waterbody No. 6 is located in the centre of the small village of Drwęsa, near a guesthouse and in the vicinity of a ground sewage treatment plant and a farm. This waterbody originated in the 1980s after a torrential rain, when water gathered in the hollow of the area. It was artificially enlarged by removing the soil. It is used for angling and recreation. Rainwater is discharged into it from the roofs of adjacent buildings on its northeastern side. Every year excessive amounts of hornwort (*Ceratophyllum demersum*) are removed from this waterbody^23^.

### Quality of water—methods of analysis

The temperature, pH, electrolytic conductivity and oxygen dissolved in water were measured in the surface layer of each waterbody using a YSI multi-parameter meter (Xylem Inc., Ohio, USA).

The total suspension was analysed with the weight method. The concentration of chlorophyll *a* was measured spectrophotometrically with the Lorenzen method (PN-86-C-05560/02). Ammonium nitrogen was analysed with the method using Nessler’s reagent (PN-C-04576-4), nitrate nitrogen—with sodium salicilate (PN-82 C-04576.08), nitrite nitrogen—with sulphanilic acid and 1-naphthylamine (PN-73 C-04576), organic nitrogen—using the Kjeldahl method after mineralization in concentrated sulphuric acid with selenium mixture (PN-EN 25663), dissolved phosphates—with the method using ammonium molybdate and ascorbic acid (PN-89 C-04537), total phosphorus—in the same way after prior mineralisation (PN-91 C-04537/09)^24^. The concentrations of organic phosphorus (P_org_) were calculated.

The total organic carbon (TOC) content and dissolved organic carbon (DOC) content were measured with a SHIMADZU TOC-V CPN analyser. The colour was determined visually by comparing the water sample with the platinum-cobalt scale.

### Analysis of vegetation and sources of pollution

Every year in summer all species of plants growing in the waterbodies and along their shores were inventoried. Maps and the QGis program were used to determine and calculate their catchment areas (ha). The percentage share of basic forms of land use was estimated. The land cover of the catchment area in the buffer zones extending at distances of 100 m and 200 m away from the shoreline of the waterbodies was analysed with the ArcMap software in order to assess the influence of land management. The shortest distance of the waterbodies from potential sources of pollution was determined by means of GIS analysis. This method is increasingly often used in environmental research^25,26^.

### Statistical analysis

Statistical analyses and models were based on discriminant analysis. The analyses verified which types of land use in the catchment area of the waterbodies extending within the 100-m-wide buffer zone, the 200-m-wide buffer zone and the entire catchment area may affect the presence of macrophytes and the physicochemical parameters of water. Canonical correspondence analysis (CCA)^27^ was used to construct the model.

Stepwise discriminant analysis was applied to find which variables determined the distribution of macrophytes and the physicochemical parameters of water to the greatest extent. All the variables were assessed and then the ones which contributed to discrimination of the group on the basis of the *p* and *F* values for a particular variable to the greatest extent were included in the model. This process was repeated until the *p* value increased above 0.05 or the *F* value dropped below 2.00 for the variable under analysis.

The significance level was determined by means of the Monte Carlo permutation test (9999 permutations). All comparisons, calculations, and graphic elements were prepared with the Canoco for Windows software package and Microsoft Excel. The following tools from Canoco for Windows were used: Canoco for Windows 4.5, CanoDraw for Windows, and WCanoIMP.

## Results

### Quality of water

Waterbody No. 1 was characterised by the highest TOC values as well as the highest concentrations of ammonium nitrogen and total phosphorus (ESM Appendix 1). The concentrations of dissolved phosphates and the values of the electrolytic conductivity of water were also high. Waterbody No. 2 was characterised by higher concentrations of dissolved phosphates and total phosphorus, as well as higher chlorophyll *a* content than in the other waterbodies. In waterbody No. 3 the highest concentrations of dissolved oxygen were noted in winter and early spring. The pH of the water was slightly alkaline or clearly alkaline throughout the entire research period. Due to the high content of nitrate nitrogen (max. 15.42 mg N dm^−3^) and high concentrations of total phosphorus (max. 3.29 mg P dm^−3^) this waterbody was classified as hypereutrophic. The concentration of chlorophyll *a* in waterbody No. 3 was also very high (up to 329.31 µg dm^−3^). Waterbody No. 4 had the highest temperature of water among the ponds under analysis. The concentrations of chlorophyll *a* increased in the second year of the research. Waterbody No. 5 was characterised by the highest values of the electrolytic conductivity and relatively high concentrations of ammonium nitrogen. Waterbody No. 6 was characterised by the lowest concentration of ammonium nitrogen, TOC, DOC, as well as the lowest values of the electrolytic conductivity of water.

### Floristic diversity

A total of 115 plant taxa were identified in the waterbodies. There were 75 species of herbaceous plants, 25 species of trees and shrubs, and 15 species of aquatic macrophytes. The highest species diversity was found in waterbody No. 6—51 taxa; the lowest—in waterbody No. 1—17 species. The number of plant taxa in the other waterbodies ranged from 26 to 36.

Most of the 15 taxa of aquatic macrophytes were found in waterbody No. 6, i.e. 9 taxa. There were no macrophytes in waterbody No. 1. Common duckweed (*Lemna minor*) was the most common species—it was found in four waterbodies. Filamentous algae were found in three waterbodies (No. 3, 5, and 6) in summer. The largest group consisted of 8 taxa, which rarely appeared in one of the two waterbodies, i.e. No. 5 or No. 6.

Herbaceous plants were always characterised by greater species diversity than aquatic macrophytes, trees, and shrubs. The variability in the number of tree and shrub species as well as aquatic macrophytes between the years of the study was not statistically significant. In 2015, the number of herbaceous plants in the vicinity of waterbodies Nos. 1–4 was significantly greater than in the previous year. In waterbody No. 6 it was significantly lower in 2014 than in 2015 (ESM Appendix 2).

### Analysis of catchment area and land use

Arable land had the greatest share (69.2%) in the small catchment area of waterbody No. 1 (0.96 ha). The share of residential buildings was also high (23.7%) (Table 2). Residential areas (34.6%) and bushy lands (33.6%) had the greatest share in the catchment area of waterbody No. 2. The eastern part of the catchment area of waterbody No. 3 was occupied by arable land, whereas the northern and western parts were occupied by single-family houses and farm buildings. Arable land had the greatest share in this catchment area, i.e. 83.8%, whereas built-up areas occupied 10.3% of it. The catchment area of waterbody No. 4 was mostly occupied by arable land (91.6%) as well as buildings and paved roads (about 6.3%). The catchment area of waterbody No. 5 was also mostly occupied by arable land (95.1%) and bushy land (4.9%). The catchment area of waterbody No. 6, like that of waterbody No. 1, has changed significantly in the last decade. The shares of residential buildings and paved roads have increased and now they amount to 31.7% and 17.5%, respectively. In consequence, the share of arable land has decreased to about 42.9%.

**Table 2:** Land use in the buffer zones and in the entire catchment areas.

Water-body no.	Land use	Buffer zone 100 m		Buffer zone 200 m		Entire catchment area	
		Area	%	Area	%	Area	%
1	Arable land	6673	69.2	Lack	Lack	6673	69.2
	Wasteland	687	7.1			687	7.1
	Built-up areas	2289	23.7			2289	23.7
	Sum	9649				9649	
2	Roads	3330	12.1	4427	12.5	4427	12.5
	Arable land	2737	10.0	5097	14.5	5098	14.4
	Wasteland	15	0.1	1110	3.1	1110	3.1
	Bushy land	11,637	42.4	11,934	33.6	11,934	33.6
	Built-up areas	9073	33.1	12,277	34.6	12,277	34.6
	Green areas	642	2.4	642	1.8	642	1.8
	Sum	2743		35,487		35,488	
3	Roads	3965	7.8	8430	5.2	20,679	3.9
	Arable land	31,932	63.0	112,529	69.5	446,286	83.8
	Wasteland	2512	4.9	2512	1.6	3067	0.6
	Built-up areas	12,308	24.3	38,552	23.8	54,788	10.3
	Bushy land	–	–	–	–	613	0.1
	Orchards	–	–	–	–	342	0.1
	Green areas	–	–	–	–	4405	0.8
	Standing waters	–	–	–	–	2457	0.5
	Sum	50,717		162,023		532,637	
4	Roads	4536	9.0	13,092	10.0	45,446	4.7
	Arable land	17,458	34.5	52,354	40.1	887,732	91.6
	Flowing waters	127	0.3	1313	1.0	1665	0.2
	Orchards	–	–	–	–	3914	0.4
	Standing waters	–	–	–	–	7508	0.8
	Wasteland	44	0.1	210	0.2	7948	0.8
	Built-up areas	28,466	56.2	63,489	48.7	15,146	1.6
	Sum	50,631		130,458		969,359	
5	Arable land	19,058	90.5	34,960	94.6	44,468	95.1
	Bushy land	1999	9.5	1999	5.4	2266	4.9
	Sum	21,057		36,959		46,734	
6	Roads	3542	15.5	4525	17.5	4525	17.5
	Arable land	9975	43.6	11,054	42.9	11,054	42.9
	Orchards	679	3.0	684	2.7	684	2.7
	Wasteland	289	1.3	892	3.4	892	3.5
	Built-up areas	7886	34.5	8164	31.6	8164	31.7
	Standing waters	479	2.1	479	1.9	478	1.9
	Sum	22,850		25,797		25,797	

**Figure 2:**
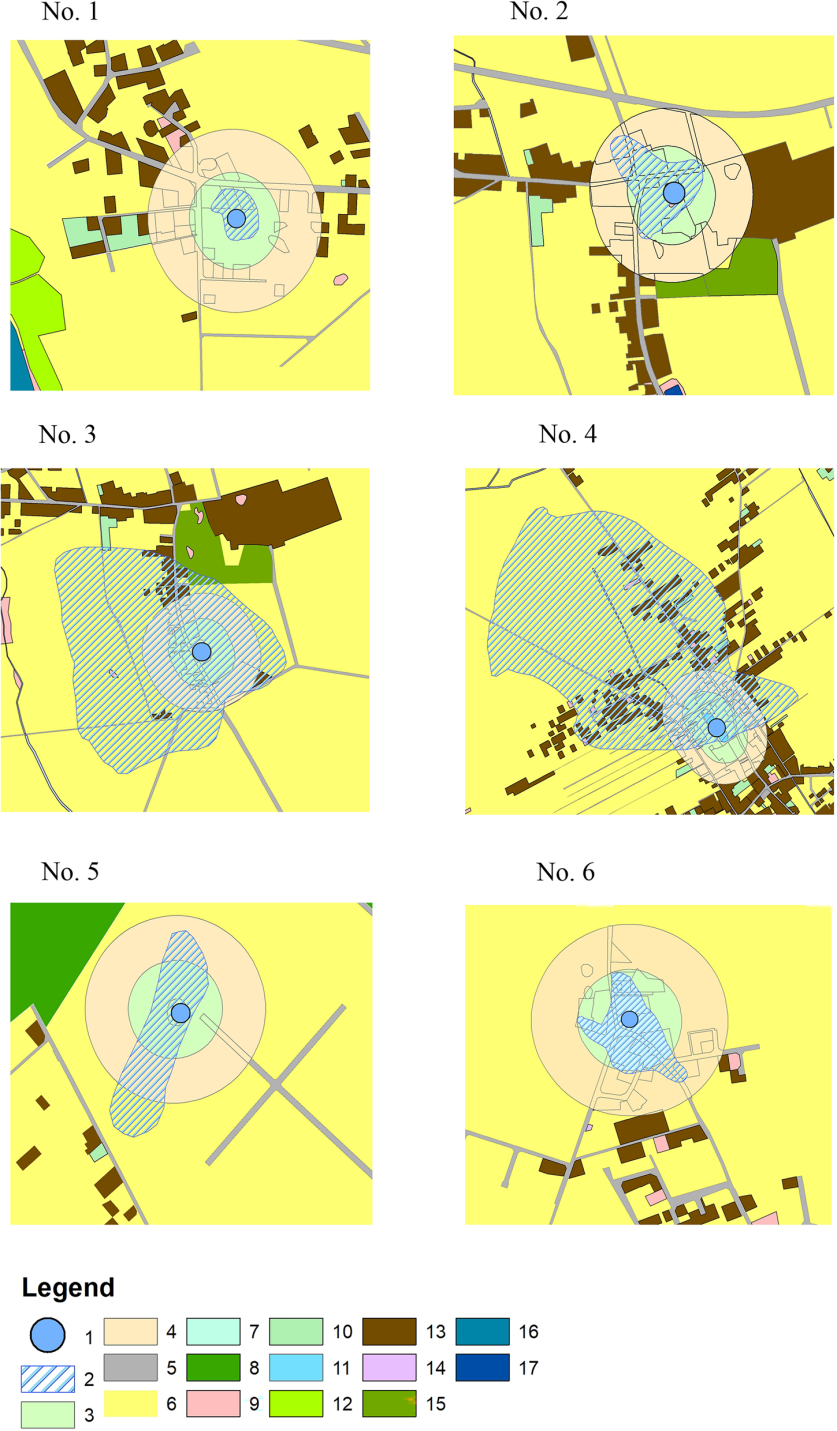
Land use in the 100- and 200-m buffer zones in the catchment areas. The map was generated by ArcGIS 10.6. https://support.esri.com/en/Products/Desktop/arcgis-desktop/arcmap/10-6-1#overview. Explanations: 1—waterbodies, 2—catchment, 3—100 m buffer zone, 4—200 m buffer zone 5—roads, 6—arable land, 7—bushy land, 8—forests, 9—wasteland, 10—orchards, 11– ponds, 12—meadows, 13—built-up areas, 14—industrial areas, 15—green areas, 16—flowing waters, 17—standing waters.

The analysis of land use in the catchment areas (Table 2, Fig. 2) within the buffer zones extending at distances of 100 m and 200 m from the waterbodies showed that arable land had the greatest share in these zones surrounding waterbodies No. 3, 5, and 6, i.e. 43.6–90.5% in the 100-m buffer zone and 42.9–94.6% in the 200-m buffer zone. Built-up areas occupied the largest parts of the buffer zones surrounding waterbodies Nos. 2 and 4—33.1–56.2% in the 100-m buffer zone and 34.6–48.7% in the 200-m buffer zone, although, as results from Table 2, arable land occupied the largest part of the entire catchment area of waterbody No. 4, i.e. 91.6%. Roads also had a significant share in both buffer zones (up to 15.5% in the 100-m buffer zone and up to 17.5% in the 200-m buffer zone). The share of bushy land in the 100- and 200-m buffer zones is up to 42.4% (in the 100-m buffer zone) and ranges from 5.4 to 33.6% (in the 200-m buffer zone), depending on the waterbody. The entire catchment area of waterbody No. 1 (0.96 ha) lies within the 100-m buffer zone. Its largest part was occupied by farmland (69.2%), whereas the smallest part was occupied by wasteland (7.1%).

### The influence of the catchment area on the composition of plants in the waterbodies and their immediate surroundings, and physicochemical variables of water quality

**Figure 3:**
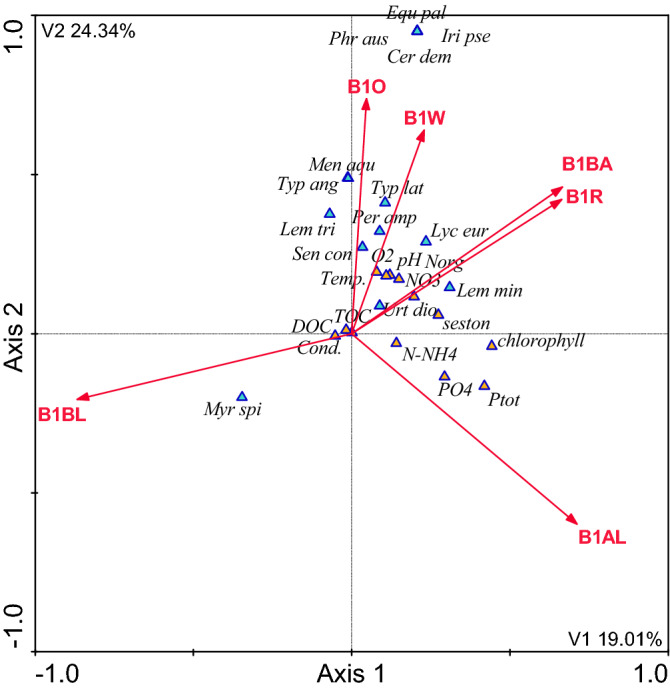
CCA model of dependencies between plant species, the physicochemical variables and land use types in the 100 m buffer zone [*Myr spi*—*Myriophyllum spicatum; Lem min*—*Lemna minor; Lem tri*—*Lemna trisulca; Urt dio*—*Urtica dioica; Sen con*—*Senecio congestus; Lyc eur*—*Lycopus europaeus; Men aqu*—*Mentha aquatica; Typ lat*—*Typha latifolia; Typ ang*—*Typha angustifolia; Per amp*—*Persicaria amphibia; Phr aus—Phragmites australis; Cer dem—Ceratophyllum demersum; Iri pse—Iris pseudacorus; Equ pal—Equisetum palustre; *DOC—dissolved organic carbon; TOC—total organic carbon; Cond.—conductivity; N-NH4—ammonium nitrogen; PO4—dissolved phosphates; Ptot—total phosphorus; NO3—nitrate nitrogen; Norg—organic nitrogen; O2—dissolved oxygen; Temp. —temperature; B1—100 m buffer zone; B2—200 m buffer zone; R—roads; AL—arable land; BL—bushy land; W—wasteland; O—orchards; FW—flowing waters; BA—built-up areas; IA—industrial areas].

**Figure 4:**
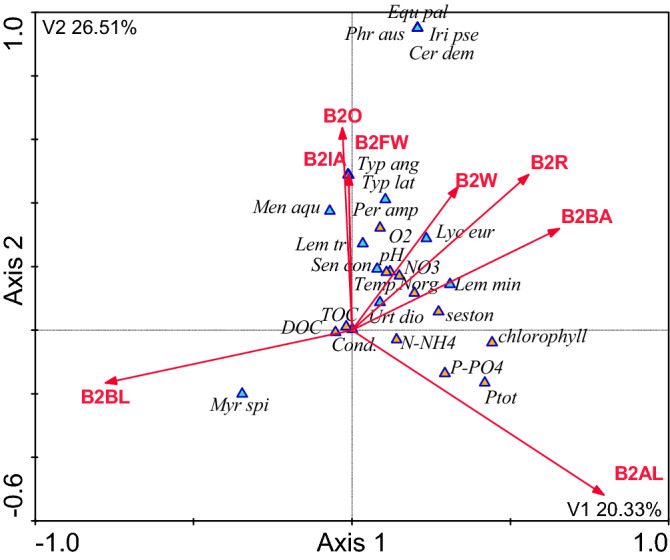
CCA model of dependencies between plant species, the physicochemical variables and land use types in the 200 m buffer zone [Explanations—see Fig. 3].

**Figure 5:**
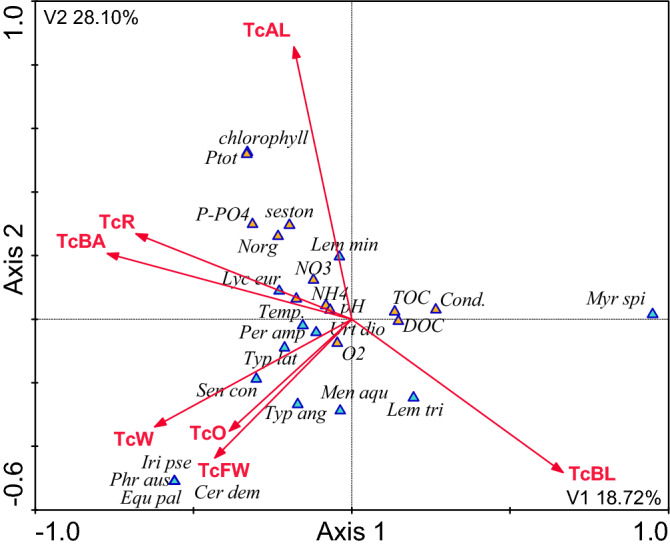
CCA model of dependencies between plant species, the physicochemical variables and land use types in the total catchment areas [*TcAL* arable land, *TcBL* bushy land, *TcBA* built-up areas, *TcR* roads, *TcW* wasteland, *TcFW* flowing waters, *TcO* orchards. Other explanations—see Fig. 3].

The analysis of the influence of land use in the entire catchment areas as well as in the 100- and 200-m buffer zones on the composition of plants in the waterbodies and their vicinity, as well as the physicochemical parameters of water quality showed that this influence was much greater in the immediate surroundings of the waterbodies than in the entire catchment areas (Figs. 3, 4, 5, Table 3). The land use of the catchment areas in the 100- and 200-m buffer zones of the waterbodies explained 67% of the variability of the parameters under analysis, but only 57% of the variability in the entire catchment areas. The following species were predominant in the waterbodies which were mostly surrounded by wasteland and orchards: *Equisetum palustre*, *Phragmites australis*, *Iris pseudacorus*, and *Ceratophyllum demersum*. The waterbodies located in the vicinity of arable land, built-up areas, and roads had higher concentrations of nutrients than those with other forms of land use in their catchment areas. The most common plant species in the waterbodies located in the vicinity of built-up areas and roads were: *Urtica dioica*, *Lycopus europaeus*, and *Lemna minor*. *Myriophyllum spicatum* was the most common species in the waterbodies surrounded by bushy land (ruderal sites) (Fig. 5, Table 3).

**Table 3:** Correction coefficients for CCA model for the buffer zone of 100 m, 200 m, total catchment and type.

Description of the code	Variable	p-value	F-value	[%] Expl
**B1 the buffer zone of 100 m**				
AL—arable land	B1AL	0.001	11.81	18.35
BL—bushy land	B1BL	0.001	9.22	15.23
O—orchards	B1O	0.002	8.91	11.33
BA—built-up areas	B1BA	0.003	8.27	9.00
R—roads	B1R	0.011	6.01	7.81
W—wasteland	B1W	0.027	4.19	5.57
**B2 the buffer zone of 200 m**				
AL—arable land	B2AL	0.001	7.52	11.22
BL—bushy land	B2BL	0.001	6.85	10.41
R—roads	B2R	0.002	6.25	9.55
BA—built-up areas	B2BA	0.003	5.44	8.51
O—orchards	B2O	0.007	5.12	7.99
FW—flowing waters	B2FW	0.015	4.77	7.23
W—wasteland	B2W	0.025	4.21	6.50
IA—industrial areas	B2IA	0.041	3.98	5.94
**Tc for the total catchment area**				
AL—arable land	TcAL	0.001	5.21	10.15
BL—bushy land	TcBL	0.002	5.01	9.36
BA—built-up areas	TcBA	0.012	4.87	8.54
R—roads	TcR	0.015	4.66	8.00
W—wasteland	TcW	0.027	4.27	7.25
FW—flowing waters	TcFW	0.031	3.95	7.04
O—orchards	TcO	0.045	2.89	6.49
**The type of waterbody**				
Total organic carbon	TOC	0.001	11.32	8.79
Dissolved phosphates	PO4	0.002	9.89	7.56
Dissolved oxygen	O2	0.011	7.77	7.21
Temperature	Temp	0.034	3.77	6.10
Ammonium nitrogen	NH4	0.037	2.23	5.11
Nitrate nitrogen	NO3	0.051	1.92	4.91

In each case arable land exerted the greatest influence. It was the greatest when fields were located up to 100 m from the shoreline—18.35% of the explained variability. This influence decreased to almost a half of this value, i.e. 10.15%, in the entire catchment areas as the distance from the waterbodies increased. Bushy land was the second most influential indicator of land use in the catchment area, explaining 9.36–15.23% of the variability of the parameters under study. Orchards in the 100-m buffer zone were the third most influential indicator, explaining 11.33% of the variability. Roads in the 200-m buffer zone explained 9.55%, whereas built-up areas explained 9.00–8.51%. Running water, wasteland, and industrial areas were also statistically significant, but these factors explained the variability of the indicators to a much lesser extent (Tables 2, 3).

**Figure 6:**
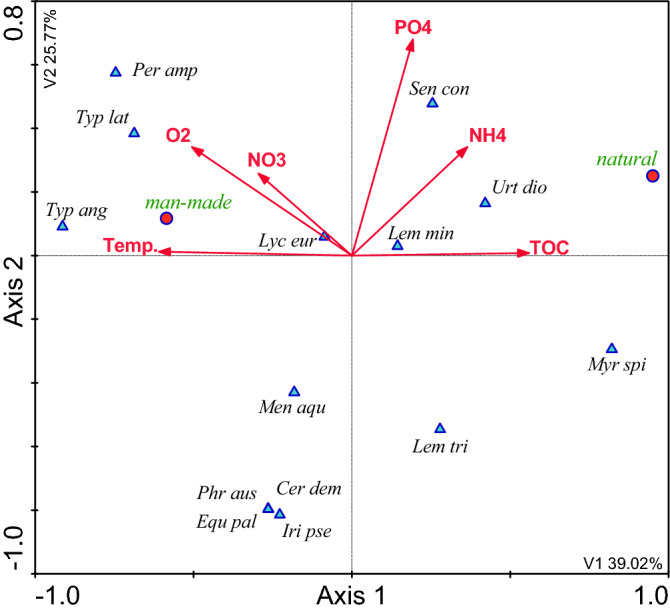
CCA model of dependencies between plant species, the physicochemical variables and type of waterbody.

The comparison of the natural and artificial waterbodies revealed big differences in the physicochemical composition of their waters and the macrophytes growing in them. The water in the natural waterbodies had higher TOC (max. 49 mg C dm^−3^ in waterbody No. 1), NH_4_ (max. 7.69 mg N dm^−3^ in waterbody No. 1), and PO_4_ (max. 3.95 mg P dm^−3^ in waterbody No. 2) concentrations. The following macrophytes were the most common in these waterbodies: *Urtica dioica*, *Senecio congestus*, *Lemna minor*, and partly *Myriophyllum spicatum*. The water in the artificial waterbodies was characterised by higher content of oxygen (max. 28.30 mg O_2_ dm^−3^ in waterbody No. 4), higher temperature (max 29 °C in waterbody No. 4), and higher concentration of nitrates (max. 15.42 mg N dm^−3^ in waterbody No. 3) in the annual cycle. The following species were predominant in these waterbodies: *Typha angustifolia*, *T. latifolia*, *Persicaria amphibia*, and *Lycopus europaeus* (Fig. 6, Table 3).

### Analysis of the distance of the waterbodies from potential sources of pollution

The waterbodies were also analysed for the distance from the nearest potential sources of pollution (Table 4). The shortest distances from livestock farms, sand and gravel mines, sewage treatment plants, landfills, industrial plants, and roads were determined. The analysis showed that the shortest distance between a farm and a waterbody was 20 m (waterbody No. 2), whereas the longest distance was 4,281 m (waterbody No. 4). During the two years of the research the trophic state of waterbody No. 2 was deteriorating gradually, which may have been caused by the proximity of a pig farm. Apart from waterbody No. 5, all the other waterbodies were located at a relatively short distance from roads (22–51 m), which may cause serious linear pollution after each heavy rainfall. There were no industrial buildings within the 200-m buffer zone of any of the waterbodies. Waterbody No. 2 was at the shortest distance from industrial buildings (418 m), but they were located outside its catchment area. Other potential sources of pollution were located much further away, i.e. at a distance of 2.5–8.33 km, and they posed minimal risk.

**Table 4:** The shortest distance from potential sources of pollution.

Waterbody no.	Distance from nearest livestock farm (m)	Distance from nearest mine (m)	Distance from nearest sewage treatment plant (m)	Distance from nearest landfill (m)	Distance from nearest industrial plant (m)	Distance from nearest road (m)
1	1179	7973	7133	7087	966	51
2	20	6836	6720	6698	418	32
3	418	7000	6200	6174	931	33
4	4281	7040	2497	2538	897	26
5	3779	4596	4292	4688	2416	243
6	1780	7998	8280	8239	1301	22

## Discussion

Aquatic vegetation, especially submerged plants, are strongly influenced by the quality of water. In highly eutrophic waterbodies with high concentrations of nutrients phytoplankton is the dominant primary producer, which causes the so-called turbid state. In mesotrophic and poorly eutrophicated waterbodies, light penetrates through water to the bottom, thanks to which macrophytes can grow. Competition for nutrients and excretion of metabolites (allelopathy) prevent the multiplication of phytoplankton, thanks to which waterbodies have clear water^7,28^. Saprotrophic waterbodies with high content of organic matter (TOC, DOC) and ammonium nitrogen are dominated by pleustophytes^23^. These interdependencies between the quality of water and macrophytes prompted the authors of this study to consider these factors jointly and assess the response of the ecosystems of small waterbodies to the influence of their catchment areas, especially the dependence between the land use in the catchment area and the increasing distance from the shoreline.

The negative influence of the catchment area mainly consists in supplying excessive loads of nutrients to waterbodies, which accelerate their eutrophication^15,29^. Every year the amount of pollution from the catchment area increases, mainly due to the intensification of agricultural production^30,31^. These pollutants include both mineral fertilisers and sewage of animal origin, including slurry from smaller farms and liquid manure from large livestock farms. Therefore, the level of animal husbandry significantly influences the amount of pollution in the area^32^. As a result, waterbodies become degraded and they disappear as a result of silting and overgrowing^33^.

The canonical correspondence analysis (CCA) confirmed the fact that farming, especially the part of the catchment area occupied by farmland, had the greatest influence on the quality of water and vegetation in small waterbodies. The analysis also showed that arable land located in the buffer zone extending up to 100 m from the shoreline exerted the greatest influence. As the distance increased, this influence weakened noticeably. This effect might be related to the retention of nutrients in soil, which is mainly caused by their sorption complex. During the growing season the uptake and retention of nutrients by crops is also important. Dense vegetation in arable fields also limits water and wind erosion in summer []20]. The CCA models showed that the influence of fields (F value for B1 = 11.81 and B2 = 7.52—Table 3) was manifested by high concentrations of phosphates (max. 3.95 mg P dm^−3^), total phosphorus (max. 5.85 mg P dm^−3^), ammonium nitrogen (max. 7.69 mg N dm^−3^), and chlorophyll *a* (752.66 μg dm^−3^), which pointed to high eutrophication of these waterbodies. At the same time, the waterbodies lacked vegetation. Their waters were turbid and dominated by phytoplankton. The analysis of the influence of the entire catchment areas showed that if the waterbodies were strongly influenced by the vicinity of fields, *Lemna minor* was the predominant species. This pleustophyte is commonly found in waters with high concentrations of nutrients, such as small shaded field ponds, which indicates their very high trophic state^34,35^. According to Szpakowska et al.^23^, waterbodies with *Lemna minor* should be classified as saprotrophic due to the high content of organic matter in the water.

Bushy land was the second indicator of land use in the catchment area explaining a large part of variability in the water quality and macrophyte composition. In the CCA models this indicator was opposite to arable land, which points to a negative correlation between these factors. Bushy land is a very important factor limiting the influence of the catchment area on waterbodies, because humans do not have direct influence on these areas. This observation is consistent with numerous scientific reports on buffer strips and biogeochemical barriers^36–38^. There are usually deeply-rooted trees growing in buffer strips. They take up large amounts of water and nutrients. There were no forests in the catchment areas of the small waterbodies analysed in our study. Some of them had only individual trees growing along the shoreline (Nos. 1, 2, 3, and 6). However, our analysis showed that bushy areas also perfectly protected the waterbodies from the inflow of spatial pollution. This fact was evidenced by the low concentrations of nutrients (min. NO_3_ 0.06 mg N dm^−3^, min. PO_4_ 0.08 mg P dm^−3^) and chlorophyll *a* (min. 2.89 μg dm^−3^) in the water and the presence of *Myriophyllum spicatum*. This is a typical example of clear-water waterbodies^7^. *M. spicatum* stabilises good quality of water because this species intensely releases allelopathic compounds^28,39^. Apart from that, submerged vegetation, including *M. spicatum*, is a refugium for crustacean zooplankton, especially large cladocerans, which feed on phytoplankton and increase water transparency^10^.

The CCA models showed that orchards and wasteland played a similar role to that of bushy areas. It is noteworthy that the orchards in the catchment areas under study were neither large nor highly productive. These were home orchards used for extensive fruit production. Therefore, their influence was similar to that of buffer strips covered with trees. The wasteland in the catchment areas was covered with perennial vegetation, with a high share of grassed strips. According to Lacas et al.^40^, they can be an important barrier protecting waters from pollution transmitted by surface runoff from farmland. In our study the protective role of orchards and wasteland was confirmed by the good oxygenation of water, its alkaline pH, and above all, by the high diversity of macrophytes, mainly helophytes, but also *Persicaria amphibia* and the submerged species of *Ceratophyllum demersum*. Although these species are typical of eutrophic waterbodies, they cannot be found in hypereutrophic ones, which have almost no vegetation at all. These species can be frequently found in agricultural landscape in eutrophic waterbodies, but with a more diversified spatial structure^35,41^.

Built-up areas and roads had a different role. In CCA models they were negatively correlated with bushy areas. This means that these forms of land use in the catchment areas had negative influence on the quality of water and the composition of macrophytes in small waterbodies. The influence of built-up areas and roads occupied an intermediate position between the influence of farmland and the influence of orchards and wasteland. The influence of built-up areas and roads was manifested by the strongly eutrophic nature of waterbodies, with high concentrations of nitrates, organic nitrogen, and seston, with a small diversity of macrophytes. Due to the fact that built-up areas and roads had a significant share in the 100- and 200-m buffer zones *Lemna minor* was the predominant species in the waterbodies, which indicated the saprotrophic nature of the habitat^23^. Rural built-up areas mostly have farm buildings. They may exert stronger negative influence on the vegetation of small waterbodies than farmlands^4^. The analysis of the distance between the waterbodies and farms showed that waterbody No. 2 was the closest to a livestock farm (20 m). The farm produced mainly pigs and poultry as well as crops and animal feed. Its location in the 100-m buffer zone may strongly deteriorate the quality of water due to both surface runoff after heavy rains and the penetration of pollutants into groundwater. Slurry is often considered the main source of environmental pollution^42^. Nitrogen and phosphorus compounds from livestock manure may be a serious problem if slurry from large farms is not properly stored and handled. Some farms do not have enough area in fields to use slurry for agricultural purposes. Large farms cooperate with other farmers to handle their slurry, which often involves numerous abuses^43^. As results from the Agriculture report^44^, the slurry handling method is mostly affected by the desire to reduce costs rather than the will to optimise fertilisation or by the actual needs of the habitat.

The presence of both buildings and roads involves the hardening of the surface of the catchment area. Impermeable surfaces generate surface runoff, which is particularly intense after heavy rainfall. Water flowing from impervious surfaces in catchment areas contains large amounts of suspensions, including organic ones, as well as large amounts of nutrients, heavy metals, and other pollutants^45–47^. This has particularly negative influence on the ecosystem of waterbodies, especially on benthic macroinvertebrates^48,49^ and macrophytes^50,51^. There were roads running at a short distance from nearly all of the waterbodies under study (except No. 5). They had significant influence on the quality of water and macrophytes. The CCA showed that the influence of roads in the 200-m buffer zone was greater than in the 100-m zone (9.55% and 7.81% of the explained variability, respectively). This means that contaminated water easily moves over paved surfaces at relatively long distances, unlike the penetration of pollutants from farmland. This is an important indication for rainwater management, because there are usually no storm drain systems in rural areas. In such cases it is important to drain rainwater from roads and let it seep into the groundwater. This also applies to other impervious surfaces within the catchment area. It is advisable to replace concrete and asphalt surfaces with openwork structures, which allow water to soak into the ground.

The concept of biogeochemical barriers is gaining importance in agricultural landscape because they may counteract the spread of pollutants carried with surface water and groundwater. As results from the analysis conducted in our study, not only areas with trees can play the role of such buffer strips, but these can also be bushy areas, orchards used for extensive fruit production, areas with perennial herbaceous vegetation (e.g. wastelands), and waterbodies. The essence of their influence is the ability to capture chemicals dissolved in water and accumulate them in plant biomass or in litter and soil within buffer strips as well as in sediments in waterbodies, where they are biochemically transformed by communities of organisms. Perennial vegetation around and in the waterbody plays a special role in the capturing of pollutants and nutrients contained in fertilisers, which are leached from fields^37^. Buffer strips, especially those with trees, also significantly affect carbon sequestration, both in plants and in soil^38^. Buffer zones are the places where plants assimilate inorganic compounds, including nitrogen and phosphorus. Soil microorganisms in buffer zones are involved in biogeochemical processes, whereas soluble and insoluble phosphorus compounds are sorbed and transported. The presence of diverse vegetation such as trees, shrubs, and grasses within this habitat results in intense removal of nutrients from waters migrating from the catchment area to the waterbody.

Changes in the land use structure, especially in the immediate vicinity of waterbodies, are particularly important in small catchment areas, because allochthonous substances often enter waters from their entire area^12^. Due to the ability to retain water by small waterbodies, the amounts accumulated during periods of its excess, e.g. during the thaw period, can be used during the growing season, because it can be accessed by plants^3^. As buffer strips and the vegetation of waterbodies are characterised by greater evapotranspiration than the vegetation of arable fields, they improve the microclimate and counteract the effects of drought^52^. Waterbodies are an important element in the water cycle, as they significantly influence the water balance in agricultural catchment areas and water relations in soils^15^.

It is possible to effectively counteract the degradation of small aquatic ecosystems in rural areas by increasing the area occupied by diverse, permanent vegetation (trees, shrubs, perennial vegetation) in the buffer zone extending within 100 m from the shoreline in order to separate farmland, built-up areas, and roads from waterbodies. It would be advisable to move intensive farming production beyond the entire 100-m-wide buffer zone, or at least reduce the intensity of agricultural activity.

## Summary

The catchment areas of waterbodies significantly affect small aquatic ecosystems. As the distance from the shoreline increases, the influence of the catchment area decreases. The amount of pollutants flowing into waterbodies mostly depends on the type of land use in the catchment area.

The presence of bushy areas, orchards, and wasteland with perennial vegetation in the vicinity of small waterbodies is particularly important in the agricultural landscape. They protect the water in the waterbodies from diffuse pollution. It is possible to accelerate or inhibit the inflow of mineral and organic matter to the waterbodies by using different forms of spatial management in the 100-m buffer zone. The right landscape structure, stimulating the intensity of a small water cycle, is also important to counteract the overdrying of the soil cover in these areas, which is one of the greatest threats to the agricultural environment. That is why it is so important to maintain small waterbodies in good ecological condition. Thanks to macrophytes, small waterbodies are characterised by high evaporation, which improves the microclimate in their vicinity. Small waterbodies are particularly important for the retention of water, because they prevent the excess of spring waters from flowing into the sea and they locally intensify the small water cycle.

The ecological approach to environmental protection requires not only the technical elimination of sources of pollution. It is also necessary to increase the resistance of natural systems to degradation by stimulating self-cleaning processes in the environment with a significant contribution of biogeochemical barriers.

## Supplementary Information

Supplementary Information 1.

Supplementary Information 2.
