# Supplementary material for: The influence of land use in the catchment area of small waterbodies on the quality of water and plant species composition
Source: Sci Rep. 2022 May 4;12:7265. doi: 10.1038/s41598-022-11115-w (PMC9068702; doi:10.1038/s41598-022-11115-w)
Supplement: Supplementary file 1 — Supplementary Information 1. [file 41598_2022_11115_MOESM1_ESM.docx]

| Waterbody No. |  | Dissolved oxygen [mg O_2_ · dm^–3^] | Dissolved oxygen [%] | Temperature [ºC] | pH value [-] | Conductivity [μS · cm^–1^] | TOC [mg C · dm^–3^] | DOC [mg C · dm^–3^] | N-NH_4_ [mg N · dm^–3^] | N-NO_3_ [mg N · dm^–3^] | P_tot_ [mg P · dm^–3^] | P-PO_4_ [mg P · dm^–3^] | N_org_ [mg N · dm^–3^] | Chlorophyll a [μg · dm^–3^] | Seston dry mass [mg · dm^–3^] |
| --- | --- | --- | --- | --- | --- | --- | --- | --- | --- | --- | --- | --- | --- | --- | --- |
| 1 | min | 5.20 | 60.00 | 18.00 | 8.11 | 1523.00 | 39.00 | 42.00 | 7.43 | 0.75 | 4.56 | 1.33 | - | 21.13 | 9.60 |
|  | max | 6.10 | 65.00 | 21.50 | 8.44 | 1741.00 | 49.00 | 44.00 | 7.69 | 0.89 | 4.67 | 1.52 | - | 55.00 | 22.70 |
|  | average | 5.67 | 62.33 | 19.83 | 8.29 | 1624.67 | 44.33 | 43.00 | 7.55 | 0.83 | 4.60 | 1.44 | - | 33.41 | 14.93 |
|  | SD | 0.45 | 2.52 | 1.76 | 0.17 | 109.74 | 5.03 | 1.00 | 0.14 | 0.07 | 0.06 | 0.10 | - | 18.76 | 6.88 |
| 2 | min | 0.30 | 3.00 | 19.50 | 6.72 | 680.00 | 13.00 | 12.00 | 1.12 | 0.34 | 1.17 | 0.36 | 0.34 | 42.17 | 9.20 |
|  | max | 3.20 | 34.00 | 22.10 | 7.69 | 740.00 | 21.00 | 19.00 | 6.18 | 1.24 | 5.85 | 3.95 | 13.32 | 752.66 | 84.00 |
|  | average | 1.50 | 16.33 | 21.03 | 7.15 | 706.67 | 18.00 | 14.33 | 2.97 | 0.71 | 2.88 | 1.63 | 5.15 | 289.78 | 38.50 |
|  | SD | 1.51 | 15.95 | 1.36 | 0.49 | 30.55 | 4.36 | 4.04 | 2.79 | 0.47 | 2.58 | 2.01 | 7.11 | 401.19 | 39.94 |
| 3 | min | 8.20 | 80.00 | 23.10 | 8.13 | 840.00 | 11.00 | 8.80 | 1.26 | 0.12 | 0.30 | 0.15 | 0.77 | 2.89 | 8.20 |
|  | max | 26.50 | 119.00 | 27.10 | 8.97 | 940.00 | 18.00 | 15.00 | 1.50 | 15.42 | 3.29 | 1.36 | 4.21 | 329.31 | 61.60 |
|  | average | 14.90 | 99.33 | 25.40 | 8.47 | 883.33 | 14.00 | 11.93 | 1.38 | 5.61 | 1.83 | 0.83 | 2.04 | 132.85 | 31.67 |
|  | SD | 10.09 | 19.50 | 2.07 | 0.44 | 51.32 | 3.61 | 3.10 | 0.12 | 8.52 | 1.49 | 0.62 | 1.89 | 173.07 | 27.28 |
| 4 | min | 7.60 | 85.00 | 23.50 | 8.52 | 740.00 | 9.10 | 7.60 | 0.85 | 0.10 | 0.21 | 0.12 | 1.03 | 43.53 | 18.40 |
|  | max | 28.30 | 182.00 | 29.00 | 8.85 | 790.00 | 17.00 | 16.00 | 1.20 | 1.60 | 0.96 | 0.51 | 2.13 | 67.18 | 30.00 |
|  | average | 17.00 | 121.33 | 26.57 | 8.70 | 756.67 | 12.70 | 11.20 | 1.06 | 1.00 | 0.59 | 0.34 | 1.48 | 58.11 | 24.13 |
|  | SD | 10.48 | 52.88 | 2.80 | 0.17 | 28.87 | 4.00 | 4.33 | 0.19 | 0.80 | 0.38 | 0.20 | 0.58 | 12.75 | 5.80 |
| 5 | min | 1.34 | 24.90 | 21.10 | 8.17 | 1930.00 | 34.00 | 31.00 | 1.29 | 0.38 | 0.10 | 0.08 | 1.40 | 3.47 | 5.40 |
|  | max | 14.30 | 167.00 | 24.80 | 9.48 | 3650.00 | 42.00 | 40.00 | 1.58 | 1.37 | 0.12 | 0.18 | 2.57 | 16.91 | 13.00 |
|  | average | 7.15 | 86.63 | 22.77 | 8.82 | 2653.33 | 39.33 | 36.00 | 1.42 | 0.88 | 0.12 | 0.12 | 1.79 | 8.27 | 9.47 |
|  | SD | 6.58 | 72.86 | 1.88 | 0.66 | 891.98 | 4.62 | 4.58 | 0.15 | 0.50 | 0.01 | 0.06 | 0.67 | 7.50 | 3.83 |
| 6 | min | 4.40 | 22.60 | 21.30 | 7.21 | 360.00 | 9.50 | 8.90 | 0.74 | 0.06 | 0.13 | 0.08 | 1.22 | 5.36 | 5.00 |
|  | max | 5.20 | 55.00 | 22.30 | 9.36 | 440.00 | 11.00 | 9.60 | 1.17 | 1.43 | 0.31 | 0.26 | 7.84 | 123.36 | 14.00 |
|  | average | 4.80 | 41.20 | 21.73 | 8.11 | 386.67 | 10.17 | 9.33 | 0.94 | 0.66 | 0.25 | 0.16 | 3.63 | 58.89 | 9.33 |
|  | SD | 0.40 | 16.72 | 0.51 | 1.12 | 46.19 | 0.76 | 0.38 | 0.21 | 0.70 | 0.10 | 0.09 | 3.66 | 59.76 | 4.51 |

Appendix 1. Minimum, maximum, mean values and standard deviation for examined parameters
