# Supplementary material for: The influence of land use in the catchment area of small waterbodies on the quality of water and plant species composition
Source: Sci Rep. 2022 May 4;12:7265. doi: 10.1038/s41598-022-11115-w (PMC9068702; doi:10.1038/s41598-022-11115-w)
Supplement: Supplementary file 2 — Supplementary Information 2. [file 41598_2022_11115_MOESM2_ESM.docx]

Appendix 2.. The number of plant taxa in waterbodies no. 1-6 and their immediate vicinity within two years of research 2014 and 2015 (The letters indicate statistically significant differences)

| Waterbody | Plant taxa | 2014 | 2015 |
| --- | --- | --- | --- |
| No. 1 | Trees and shrubs | 3 a | 3 a |
|  | Herbaceous plants | 8 a | 14 b |
|  | Aquatic macrophytes | 0 a | 0 a |
| No. 2 | Trees and shrubs | 8 a | 8 a |
|  | Herbaceous plants | 6 a | 9 b |
|  | Aquatic macrophytes | 1 a | 1 a |
| No. 3 | Trees and shrubs | 3 a | 3 a |
|  | Herbaceous plants | 9 a | 18 b |
|  | Aquatic macrophytes | 5 a | 2 a |
| No. 4 | Trees and shrubs | 1 a | 1 a |
|  | Herbaceous plants | 10 a | 22 b |
|  | Aquatic macrophytes | 2 a | 2 a |
| No. 5 | Trees and shrubs | 4 a | 3 a |
|  | Herbaceous plants | 16 a | 14 a |
|  | Aquatic macrophytes | 8 a | 7 a |
| No. 6 | Trees and shrubs | 12 a | 12 a |
|  | Herbaceous plants | 13 b | 19 a |
|  | Aquatic macrophytes | 9 a | 7 a |
